# Phylogenetic and structural analysis of centromeric DNA and kinetochore proteins

**DOI:** 10.1186/gb-2006-7-3-r23

**Published:** 2006-03-22

**Authors:** Patrick Meraldi, Andrew D McAinsh, Esther Rheinbay, Peter K Sorger

**Affiliations:** 1 Department of Biology, Massachusetts Institute of Technology, Massachusetts Ave., Cambridge, MA 02139, USA; 2 Institute of Biochemistry, ETH Zurich, Schafmattstr.,18 CH-8093 Zurich, Switzerland; 3 Chromosome Segregation Laboratory, Marie Curie Research Institute, The Chart, Oxted, Surrey RH8 0TL, UK

## Abstract

**Background:**

Kinetochores are large multi-protein structures that assemble on centromeric DNA (*CEN *DNA) and mediate the binding of chromosomes to microtubules. Comprising 125 base-pairs of *CEN *DNA and 70 or more protein components, *Saccharomyces cerevisiae *kinetochores are among the best understood. In contrast, most fungal, plant and animal cells assemble kinetochores on *CEN*s that are longer and more complex, raising the question of whether kinetochore architecture has been conserved through evolution, despite considerable divergence in *CEN *sequence.

**Results:**

Using computational approaches, ranging from sequence similarity searches to hidden Markov model-based modeling, we show that organisms with *CENs *resembling those in *S. cerevisiae *(point *CEN*s) are very closely related and that all contain a set of 11 kinetochore proteins not found in organisms with complex *CEN*s. Conversely, organisms with complex *CEN*s (regional *CEN*s) contain proteins seemingly absent from point-*CEN *organisms. However, at least three quarters of known kinetochore proteins are present in all fungi regardless of *CEN *organization. At least six of these proteins have previously unidentified human orthologs. When fungi and metazoa are compared, almost all have kinetochores constructed around Spc105 and three conserved multi-protein linker complexes (MIND, COMA, and the NDC80 complex).

**Conclusion:**

Our data suggest that critical structural features of kinetochores have been well conserved from yeast to man. Surprisingly, phylogenetic analysis reveals that human kinetochore proteins are as similar in sequence to their yeast counterparts as to presumptive *Drosophila melanogaster *or *Caenorhabditis elegans *orthologs. This finding is consistent with evidence that kinetochore proteins have evolved very rapidly relative to components of other complex cellular structures.

## Abstract 2

Analysis of centromeric DNA and kinetochore proteins suggests that critical structural features of kinetochores have been well conserved from yeast to man.

## Background

Kinetochores are eukaryote-specific structures that assemble on centromeric (*CEN*) DNA and perform three crucial functions: they bind paired sister chromatids to spindle microtubules (MTs) in a bipolar fashion compatible with chromatid disjunction; they couple MT (+)-end polymer dynamics to chromosome movement during metaphase and anaphase [1]; and they generate the spindle checkpoint signals linking anaphase onset to the completion of kinetochore-MT attachment [2]. Despite the conservation of these functions, and of MT structure and dynamics, *CEN*s in closely related organisms are highly diverged in sequence, as are *CEN*s on different chromosomes in a single organism [2,3]. The simplest known *CEN*s, those in the budding yeast *Saccharomyces cerevisiae*, consist of 125 base-pairs (bp) of DNA and three protein-binding motifs (CDEI, CDEII and CDEIII) that are present on all 16 chromosomes [4]. These short *CEN *sequences, often called 'point' *CEN*s, are structurally similar to enhancers and transcriptional regulators in that their assembly is initiated by highly sequence-selective DNA-protein interactions [5]. In contrast, *CEN *DNA in fungi such as the budding yeast *Candida albicans *and fission yeast *Schizosaccharomyces pombe*, plants such as *Arabidopsis thaliana*, and metazoans such as *Drosophila melanogaster *and *Homo sapiens*, are longer and more complex and exhibit poor sequence conservation [6-10]. These regional *CEN*s range in size from 1 kb in *C. albicans *[6], to several megabases in *H. sapiens *[8] and typically contain long stretches of repetitive AT-rich DNA. *CEN *organization is particularly divergent in nematodes such as *Caenorhabditis elegans*, which contain holocentric *CEN*s with MT-attachment sites distributed along the length of chromosomes [11]. Sequence-selective DNA-protein interactions have not been identified in regional *CEN*s and it is thought that kinetochore position is determined by a specialized chromatin domain whose formation at one site on each chromosome is controlled by epigenetic mechanisms [2,12].

A combination of genetics and mass spectrometry in *S. cerevisiae *has yielded a fairly detailed view of the composition and architecture of its simple kinetochores. *S. cerevisiae *kinetochores contain upwards of 70 protein subunits organized into 14 or more multi-protein complexes that together have a molecular mass in excess of 5 to 10 MDa [5]. *S. cerevisiae *kinetochore proteins can be assigned to DNA-binding, linker, MT-binding and regulatory functions. While 'linker protein' is used rather loosely, all linkers exhibit a clear hierarchical relationship with respect to DNA and MT-binding proteins: linker proteins require DNA binding proteins, and possibly also other linker proteins, for *CEN *DNA binding but not MTs or MT-associated proteins (MAPs).

Kinetochore assembly in *S. cerevisiae *is initiated by association of the essential four-protein CBF3 complex with the CDEIII region of *CEN *DNA. CBF3-CDEIII association then recruits several additional DNA binding proteins, including scCse4, a specialized histone H3 found only at *CEN*s (CenH3). CenH3-containing nucleosomes are thought to be core components of all kinetochores [13]. When *CEN *associated, the DNA binding subunits of *S. cerevisiae *kinetochores recruit four essential multi-protein linker complexes, the NDC80 complex (four proteins), COMA (four proteins), MIND (four proteins) and the SPC105 complex (two proteins). These complexes, in turn, recruit a multiplicity of motor proteins and MAPs to form a fully functional MT-attachment site (P De Wulf and PK Sorger, unpublished observation) [14-16].

A key question in the study of kinetochores is whether architectural features currently being elucidated in *S. cerevisiae *are conserved in higher cells. Some *S. cerevisiae *proteins have been shown to have orthologs in one or more metazoa. These metazoan orthologs include CenH3, CENP-C^Mif2^, Mis6^Ctf3/CENP-I^, Spc105^KNL-1/Kia1570^, members of the NDC80 and MIND complexes as well as MT-associated proteins such as EB1^Bim1 ^and CLIP170^Bik1^, Mad-Bub spindle checkpoint proteins and some regulatory kinases [2,17-26]. To date, however, only CenH3 and CENP-C have been carefully compared at a sequence level in a wide range of organisms [27]. Here we report a systematic analysis of sequence relationships among a set of approximately 50 fungal, plant and metazoan kinetochore proteins with the overall aim of exploring their structural and evolutionary relationships. Our analysis supports the conclusion that the four linkers at the core of *S. cerevisiae *kinetochores, the NDC80 complex, MIND, COMA, and the SPC105 complex, have been conserved through eukaryotic evolution. A subset of kinetochore proteins, perhaps 20% of the total in *S. cerevisiae*, seems to be specific to point *CEN*s, all of which are very closely related. A second set of kinetochore proteins is found only on regional *CEN*s. It appears, therefore, that all kinetochores have a single ancestor, probably based on a regional *CEN*, from which contemporary kinetochores diverged rapidly while conserving key structural features.

## Results

### Point centromeres have a common origin

**Figure 1:**
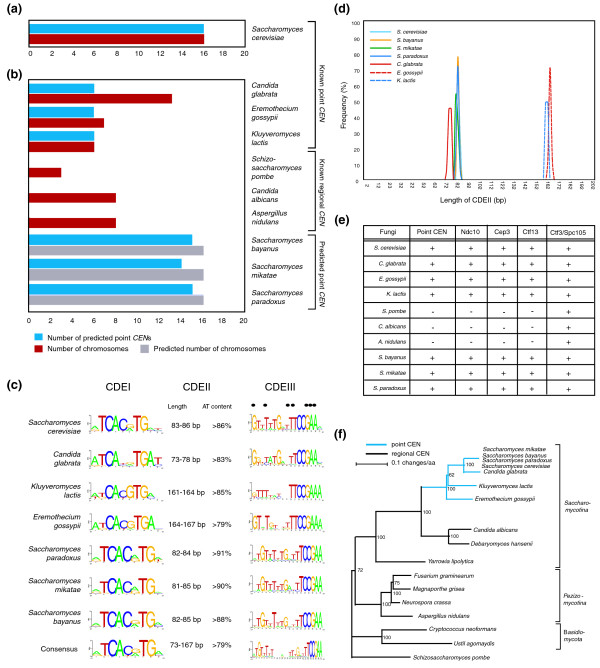
Point centromeres are derived from regional centromeres and appeared only once during evolution. **(a) **The 16 *CEN*s from *S. cerevisiae *were used to train a HMM. The blue bar indicates the number of predicted point *CEN*s in the genome and the red bar represents the number of known chromosomes. **(b) **HMM from (a) was used to search the genome of fungi with known point *CEN*s, known regional *CEN*s and predicted point *CEN*s. Blue and red bars are as described in (a) except gray bars, which indicate the predicted number of chromosomes, based on synteny within other *Saccharomyces *species. **(c) **Sequence comparison of the CDEI, CDEII and CDEIII elements from budding yeast with point centromeres. **(d) **Frequency distribution of the CDEII length (measured in bp) in each budding yeast with point centromeres. **(e) **Evolutionary conservation of CBF3 subunits in fungi with point and regional *CEN*s. **(f) **Phylogenetic analysis of 17 different fungi, including the 7 budding yeast with point centromeres and the 3 budding yeast with regional centromeres using 3 highly conserved reference proteins (α-tubulin, the signal recognition protein SRP54 and the DNA replication factor PCNA). Blue branches represent fungi with point centromeres and black branches those with regional centromeres.

As a first step in determining relationships among kinetochores in different organisms, we searched fungal genomes for point *CEN*s similar in structure to those in *S. cerevisiae*. Three such examples are already known, *C. glabrata*, *E. gossypii *and *K. lactis *[28], but a significant number of newly sequenced genomes have not yet been analyzed. Finding new *CEN*s with a CDEI-CDEII-CDEIII structure is not trivial because the number of identical bases in CDEI and CDEIII is relatively small, even among chromosomes in *S. cerevisiae*. Moreover, CDEII is not conserved in sequence but, rather, is characterized by high AT content and alternating runs of poly-A and poly-T. To capture this information we constructed a tri-partite computational model based on profiles for CDEI and CDEIII, a hidden Markov model (HMM) for CDEII (Figure 1a), and *S. cerevisiae CEN*s as a training set. When the model was tested on *C. glabrata*, *E. gossypii *and *K. lactis*, organisms whose genomes are fully annotated, 6/13 centromeres in *C. glabrata*, 6/7 centromeres in *E. gossypii *and 6/6 in *K. lactis *were identified correctly (Figure 1b). Conversely, no point-*CEN *sequences were found in *S. pombe*, *C. albicans *or *A. nidulans*, organisms known to have regional *CEN*s (Figure 1b). With a success rate of >70% and a false positive rate of <5%, we conclude that our computer model is effective at finding point *CEN*s.

When unannotated genomes were analyzed using the tri-partite computational model, 15 CDEI-II-III sequences were found in *S. bayanus*,14 in *S. mikatae *and 15 in *S. paradoxus *(Figure 1b) [29]. *S. bayanus*, *S. mikatae *and *S. paradoxus *contigs have not yet been fully assembled, but sequence similarity and synteny suggest that all 3 have 16 chromosomes, close to the number of putative *CEN *sequences identified computationally in each organism. When these newly identified point *CEN*s were combined with those in the literature, 85 CDEI-II-III sequences from 7 organisms became available. These yielded a clear consensus for CDEI and CDEIII and revealed that, within a single organism, CDEII can vary in sequence from one chromosome to the next but that length distributions are very narrow (± 3%; Figure 1c, d). Most fungi have 84 bp CDEII sequences but *E. gossypii *and *K. lactis *have 164 bp CDEIIs, suggesting the presence of two copies of an underlying approximately 84 bp CDEII module (Figure 1d). To a first approximation, the extent of conservation among CDEI and CDEIII sequences on different chromosomes within a single organism was not much greater than the extent of conservation among syntenic *CEN*s in different organisms (Figure 1c). Together, these data strongly imply that all organisms with CDEI-II-III point *CEN*s arose from a relatively recent common ancestor.

### Kinetochore proteins specific to organisms with point centromeres

Does the existence of *CEN*s with similar CDEI-II-III structures imply the existence of similar DNA-binding kinetochore proteins? In addressing this question, the CDEI-binding Cbf1 protein is not very useful because it functions not only as a kinetochore subunit but also as a transcription factor for a set of highly conserved biosynthetic genes [30], implying conservation of non-kinetochore function. We therefore concentrated on components of the CBF3 complex, three of whose subunits are thought to function only in CDEIII-binding (the fourth subunit, scSkp1, is also a component of the SCF ubiquitin ligase complex [31] and, like Cbf1, has conserved non-kinetochore functions). When PSI-BLAST was used to search predicated open reading frames in 17 fungal genomes for orthologs of scCtf13, scCep3 and scNdc10, all 3 CBF3 subunits were found in the organisms with point *CEN*s (7 in total), but not in organisms with regional *CEN*s (Figure 1e). As a positive control for the PSI-BLAST search, orthologs of scMis6^Ctf3 ^and scSpc105 could be found in all fungi examined (Figure 1e). Importantly, Mis6^Ctf3 ^and Spc105 have approximately the same degree of sequence divergence in point-*CEN *containing fungi (51% and 48% similarity, respectively) as Ndc10 (48% similarity; Table 1). We provisionally conclude that CBF3 proteins are present only in fungi with CDEI-II-III *CEN *DNA whereas other kinetochore proteins (such as Spc105 and Ctf3) are ubiquitous. Moreover, when organisms with point *CENs *and CBF3 subunits are mapped on a phylogenetic tree (constructed using the highly conserved reference proteins α-tubulin, the signal recognition particle subunit SRP54 and PCNA) they were found to cluster closely together (Figure 1f). While recognizing the possibility for false-negative findings in cross-species sequence searching, we conclude that CDEI-II-III *CEN*s and CBF3 *CEN*-binding proteins are probably found only in a subset of closely related budding yeasts and, thus, may have co-evolved. Intriguingly, the apparent common ancestor of point-*CEN *and regional-*CEN *organisms appears to be a fungus containing regional *CEN*s, implying that simple point *CEN*s arose from complex regional *CEN*s and not the other way round.

**Figure 2:**
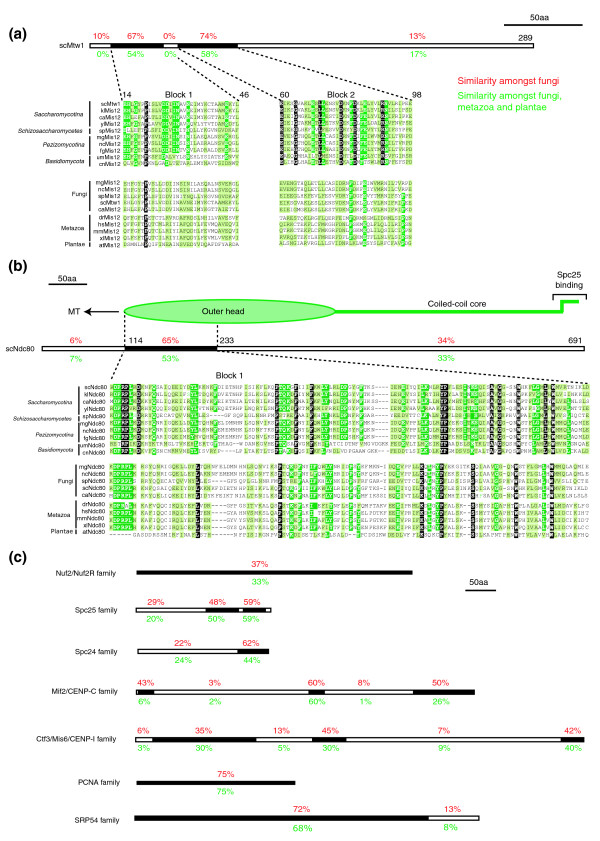
Sequence similarity between kinetochore proteins is restricted to short stretches between orthologs. Multiple sequence alignments of the **(a) **Mis12^Mtw1 ^and **(b) **Ndc80^Hec1 ^families. Schematic drawing above the alignment indicate the length of the *S. cerevisiae *proteins and the percentages denote the degree of similarity of successive sequence blocks (black boxes) within fungi (red letters) or fungi, metazoa and plantae (green letters). The schematic drawing above the Ndc80 multiple sequence alignment also indicates the relative position of the globular and coiled-coil domain of Ndc80, as determined by electron-microscopy [32,33]. White letters on black denote identical residues, white letters on green, identical residues in ≥ 80% of the organisms and black letters on green, similar residues in ≥ 80% of the organisms. **(c) **Schematic drawings indicating the percentage similarity of successive sequence blocks (black boxes) within fungi (red letters) or fungi, metazoa and plantae (green letters) based on multiple sequence alignments of the Nuf2, Spc25, Spc24. CENP-C^Mif2 ^and Mis6^Ctf3/CENP-I^, PCNA and SRP54 protein families

To delineate further which kinetochore proteins are specific to point *CEN*s, and which are more widely distributed, we analyzed all known *S. cerevisiae *kinetochore proteins for sequence conservation. As a starting point we examined scMis12^Mtw1 ^and scNdc80^Hec1^, kinetochore proteins first identified in yeast and subsequently shown to have human orthologs (hsMis12 and hsNdc80^Hec1^) that localize to kinetochores and play a role in chromosome segregation [20,25]. Experimental and sequence data establish that yeast and higher cell Ndc80^Hec1 ^and Mis12^Mtw1 ^proteins represent true orthologs [20,32-34]. Nonetheless, the overall degree of similarity among Ndc80^Hec1 ^and Mis12^Mtw1 ^proteins across eukaryotes was found to be relatively modest (approximately 15% to 30%) as compared to proteins involved in DNA replication (PCNA, approximately 75%) or protein translocation (SRP54, approximately 60%). Multiple protein sequence alignments of fungal, plant, and metazoan Ndc80^Hec1 ^and Mis12^Mtw1 ^showed that sequence similarity is confined to 30 to 100 residue blocks interspersed by stretches of non-homology, many of which correspond to coiled coils (Figure 2a, b). This pattern of block-by-block similarity was also observed with five other kinetochore proteins for which orthology has been established experimentally, and is consistent with previous proposals that kinetochore proteins have evolved rapidly [35] (Figure 2c). Importantly, for our purposes, data obtained from known kinetochore orthologs suggests that it is necessary to use conserved blocks, rather than complete sequences, when searching kinetochore proteins for patterns of sequence conservation.

**Figure 3:**
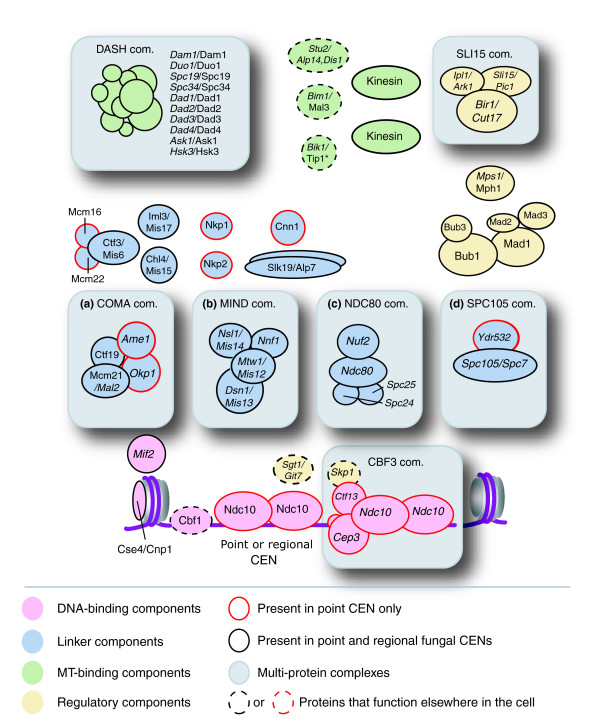
Fungal kinetochores contain a set of point centromere specific components. Schematic model of kinetochore subunitorganization based on the architecture of the *S. cerevisiae *kinetochore. Kinetochore proteins can be roughly divided into DNA-binding (pink), linker (blue), MT-binding (green) and regulatory layers (yellow). Within each layer many proteins are organized into multi-protein complexes, for example, the linker layer is composed of at least four complexes (gray boxes **(a) **to **(d)**): COMA, NDC80, MIND and SPC105. Protein names are given for *S. cervisiae *first and *S. pombe *second, while essential genes (italic letters) and non-essential (normal letters) is indicated. Protein names followed by an asterisk indicate that this specific ortholog is known not to localize to kinetochores. The kinesins present at kinetochores in *S. cerevisiae *are Kip3 (Kinesin-8), Cin8 (Kinesin-5), Kip1 (Kinesin-5) and Kar3 (Kinesin-14), while in *S. pombe *they are Klp5 (Kinesin-8), Klp6 (Kinesin-8) and Klp2 (Kinesin-14) (for nomenclature see [38].

When 55 *S. cerevisiae *kinetochore proteins (including the CBF3 subunits discussed above) were used in PSI-BLAST queries to search 14 fully annotated fungal genomes (Additional data file 1), 41 were found to have orthologs in organisms with both point and regional *CEN*s (Figure 3). These proteins included kinetochore regulators such as the Mad1-3, Bub1, BubR1/Mad3 and Mps1 checkpoint proteins and the Ipl1-AuroraB kinase, as well as many structural components. In addition to the 41 proteins mentioned above, conservation was observed for proteins such as Skp1 [31], Cbf1 [30,36] and some MAPs [37] that function at kinetochores as well as at other locations in the cell. As noted above, these proteins are likely to have been conserved for reasons other than their presence at kinetochores, and they cannot be used to infer overall similarity in kinetochore structure. In this respect, kinesin motor proteins are also difficult to analyze. Eukaryotic cells contain multiple kinesins, which are known to fall into 14 highly conserved protein families based on sequence, structure and function [38]. Typically, each kinesin has more than one cellular function and kinetochores in different organisms recruit different kinesin family members, making it difficult to determine (in the absence of experimentation) which kinesins should be considered kinetochore associated.

Leaving these complications aside, among 55 fungal kinetochore components analyzed, 11 were found in the 7 organisms with point *CEN*s and nowhere else, implying that they are specific to a CDEI-II-III *CEN *architecture (Figure 3). These 11 proteins include the CBF3 subunits scCtf13, scCep3 and scNdc10 described above, the non-essential *CNN1 *gene product, 1 subunit of the SPC105 complex (Ydr532c), two subunits of the COMA linker complex (scAme1 and scOkp1) and 4 proteins that require COMA for *CEN*-association (scMcm22, scMcm16, scNkp1 and scNkp2). Among organisms in which they are found, the 11 point *CEN*-specific proteins are as well or better conserved than ubiquitous kinetochore proteins, implying that failure to identify orthologs in more distant fungi is a consequence of their actual absence. We therefore propose that approximately 20% of the overall kinetochore in fungi containing CDEI-II-III *CEN*s is specialized to their simple *CEN*s. As expected, these specialized kinetochore subunits include proteins in direct contact with *CEN *DNA (Figure 3).

### Identification of novel human kinetochore proteins

**Figure 4:**
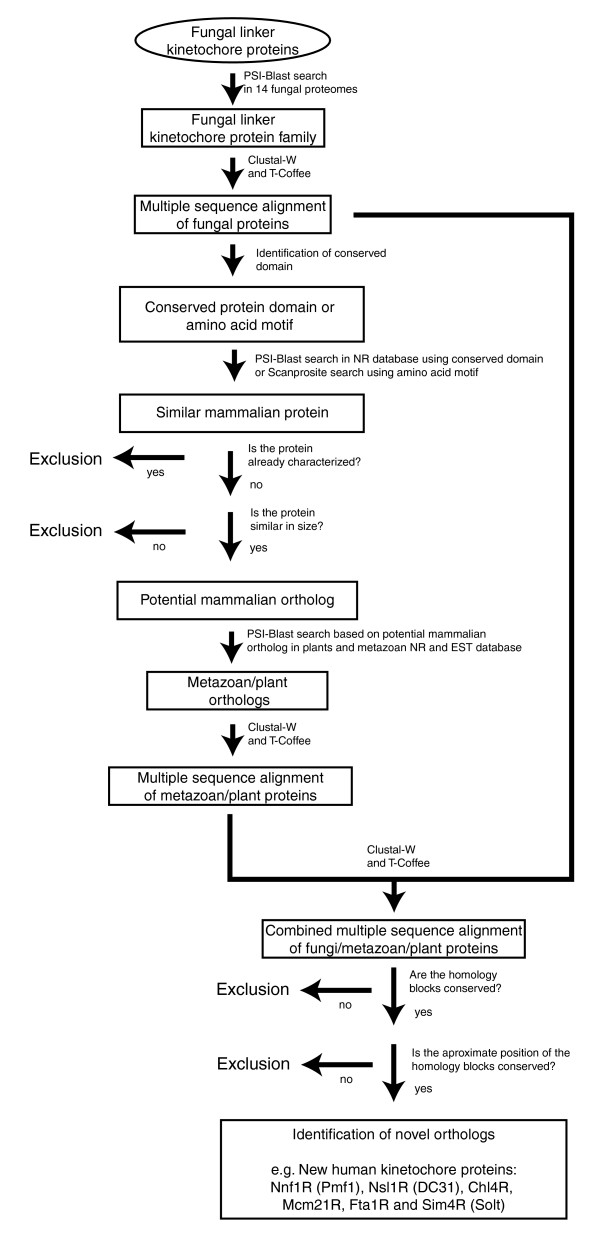
Schematic describing the sequence-search based approach used to identify fungal, metazoan, and plant orthologs of the kinetochore proteins scNnf1, scNsl1, scChl4, scMcm21, spSim4 and spFta1. Since such sequence-based searches can yield a significant number of false positives, strict exclusion criteria were applied to ensure the identification of orthologs.

Based on success in identifying fungal orthologs of *S. cerevisiae *kinetochore proteins, we expanded our set of target organisms to higher eukaryotes (see Figure 4 for a schematic of the approach). Alignments were created for 41 ubiquitous fungal proteins and conserved blocks determined. The non-redundant NCBI protein database was then searched for these conserved blocks using PSI- BLAST or Prosite pattern searching algorithms (see Materials and methods for details). Potential orthologs differing greatly in size from the fungal proteins and candidates with well-established non-kinetochore functions were eliminated from further consideration. The remaining proteins were then aligned to confirm the presence of conserved blocks. This search led to the identification, in a wide variety of organisms, of previously unreported orthologs of many *S. cerevisiae *kinetochore proteins (Additional data file 1), among which were four new human kinetochore proteins (Figure 4). Recent analysis of *S. pombe *kinetochore complexes by mass spectrometry revealed the presence of a set of proteins for which orthologs could not be found in *S. cerevisiae *[39,40]. When conserved sequence blocks from these *S. pombe *proteins were used to search the genomes of higher eukaryotes, two additional human proteins were flagged as likely kinetochore subunits (Figure 4). Regardless of which fungi contributed to the sequence blocks, the most highly conserved kinetochore subunits were invariably regulatory proteins such as the Mad and Bub checkpoint proteins and the Aurora B kinase. Structural proteins such as Ndc80^Hec1^, Nuf2, CENP-C^Mif2 ^and Mis12^Mtw1 ^were considerably more diverged.

**Figure 5:**
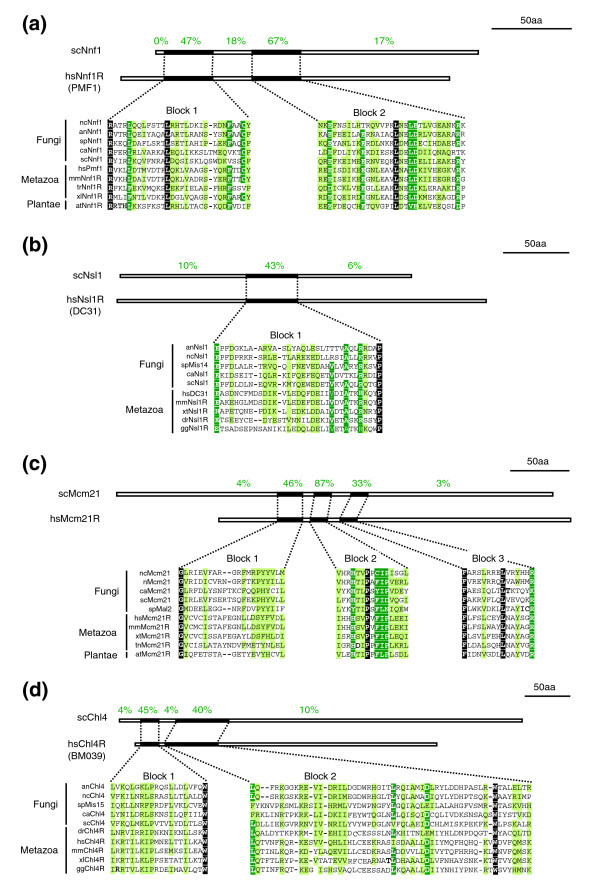
Identification of potential orthologs of scNnf1, scNsl1, scMcm21 and scChl4 in humans. *S. cerevisiae ***(a) **Nnf1, **(b) **Nsl1, **(c) **Mcm21 and **(d) **Chl4 were aligned with five fungal, four metazoan and one plant sequence. White letters on black denote identical residues, white letters on green, identical residues in ≤ 80% of the organisms and black letters on green, similar residues in ≤ 80% of the organisms. Schematic drawings above the alignments indicate the length of the *S. cerevisiae *proteins and the percentages denote the degree of similarity of successive sequence blocks (black boxes).

**Figure 6:**
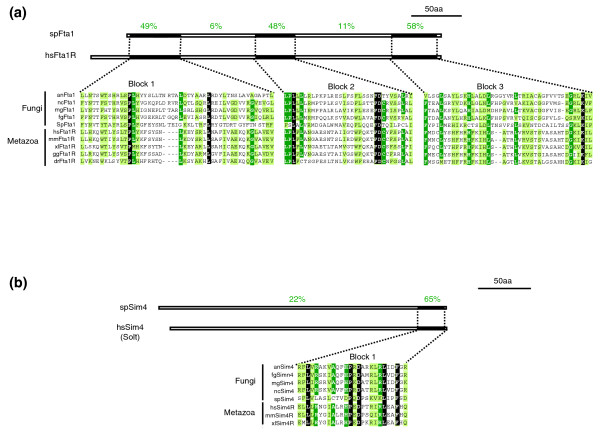
Identification of potential orthologs of spFta1 and spSim4 in humans. *S. pombe ***(a) **Fta1 and **(b) **Sim4 were aligned with five fungal, and three to five metazoan sequences. White letters on black denote identical residues, white letters on green, identical residues in ≥ 80% of the organisms and black letters on green, similar residues in ≥ 80% of the organisms. Schematic drawings above the alignments indicate the length of the *S. cerevisiae *proteins and the percentages denote the degree of similarity of successive sequence blocks (black boxes).

The four human proteins representing hitherto unrecognized orthologs of *S. cerevisiae *kinetochore subunits were provisionally named hsNnf1-Related (hsNnf1R; also known as PMF1 [41]; Figures 4 and 5), hsNsl1R (also known as DC8 or DC31), hsMcm21R and hsChl4-R. hsNnf1R shares with its fungal counterpart 2 conserved blocks of 30 to 35 residues with 47% and 67% similarity, hsNsl1R shares 1 conserved block of 35 residues with 43% similarity, hsMcm21R shares 3 conserved blocks of 15 to 30 residues with 46%, 87% and 33% similarity and hsChl4R shares 2 conserved blocks of 20 and 50 amino acids with 45% and 40% similarity (Figure 5). The potential human orthologs of *S. pombe *Fta1 and Sim4 were provisionally named hsFta1R and hsSim4R (also known as Solt [42]). hsFta1R shares with its fungal counterpart three conserved sequence blocks of 40, 25 and 30 residues with 48%, 49% and 58% similarity and hsSim4R one block of 27 residues with 65% similarity (Figure 6). Elsewhere we will describe experimental data showing that hsChl4R, hsNsl1R, hsMcm21R, hsNnf1R, hsFta1R and hSim4R localize to kinetochores in human cells and are required for accurate chromosome segregation (AD McAinsh *et al*., submitted). Importantly, for the purposes of the current analysis, the identification of new human kinetochore proteins means that one or more subunits are present in metazoans for each of the four multi-protein linker complexes forming the core of the *S. cerevisiae *kinetochore. Thus, it appears that simple point *CEN*s in budding yeast and complex regional *CEN*s in human cells probably share fundamental architectural similarities.

*S. cerevisiae *DASH is a 10-protein MT-binding complex that has attracted considerable recent interest because it forms rings encircling MTs [43,44]. DASH subunits are conserved among fungi but we have found few if any potential orthologs in higher eukaryotes. The closest match to a DASH protein in humans, NYD-SP28 [45], has an amino-terminal domain of about 30 amino acids 40% similar to *S. cerevisiae *Spc34 (Additional data file 2). The *Chlamydomonas rheinhardtii *ortholog of NYD-SP28 localizes to the flagellum [46], implying that NYD-SP28 might be involved in interactions with MTs. Our preliminary conclusion is that higher eukaryotes do not contain a protein complex closely related to fungal DASH, although further investigation of NYD-SP28 is warranted.

### Correspondence between human kinetochore proteins and their yeast counterparts

**Figure 7:**
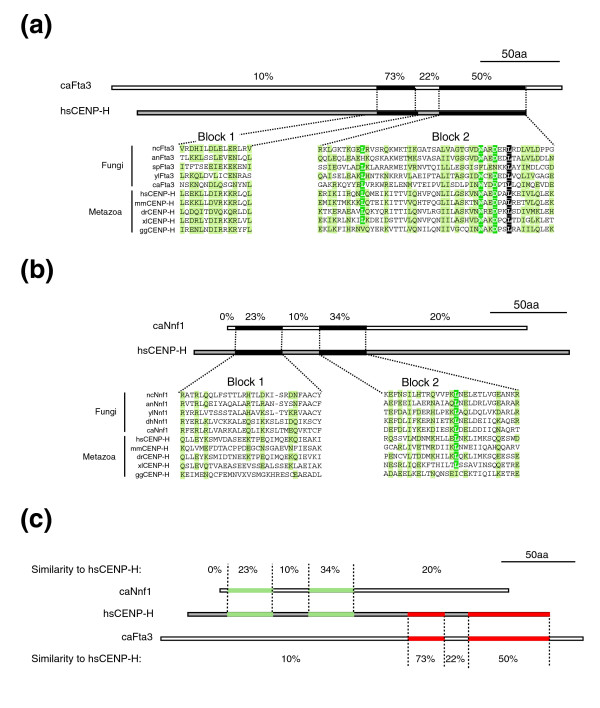
The human kinetochore protein CENP-H is more closely related to a novel family of fungal proteins than the Nnf1 family. Multiple sequence alignments of metazoan CENP-H proteins and either **(a) **fungal Fta3 family proteins or **(b) **fungal Nnf1 family of proteins. Sequences were annotated as in Figure 5. **(c) **Comparison of sequence similarity between human conserved domains of CENP-H and *C. albicans *Nnf1 and *C. albicans *Fta3.

Several kinetochore proteins first identified in human cells have previously been shown to have fungal orthologs, including CENP-C (orthologous to scMif2p [47]) and CenH3^CENP-A ^(orthologous to scCse4 [48]). We therefore wondered whether additional orthologs might be found in fungi for kinetochore proteins hitherto characterized only in higher eukaryotes, such as CENP-E, CENP-H, Rod, Zwint and Zwilch [49-53]. We found that, among fungal proteins, hsCENP-H is most similar to *S. pombe *spFta3 (Figure 7a), which was shown recently to be a fission yeast kinetochore protein [39]. It has been suggested previously that *S. cerevisiae *scNnf1 is the budding yeast CENP-H ortholog [54] (Figure 7b) but we find that scNnf1 is actually much more similar to hsNnf1R^Pmf1 ^and spNnf1 than to CENP-H (Figure 7c). We therefore propose that CENP-H is orthologous to the fungal Fta3 family of proteins. Searches using PSI-BLAST revealed that the Fta3 protein, like the Sim4 and Fta1 proteins with which it interacts in *S. pombe *[39], has apparent orthologs only in organisms with regional *CEN*s (Additional data file 1). The presence of Sim4 and Fta1 in the budding yeast *Yarrowia lipolytica*, which has regional *CEN*s, but not in yeasts with point *CEN*s, is striking, since *Y. lipolytica *is significantly closer in overall sequence to *S. cerevisiae *than to *S. pombe*. We therefore conclude that Fta3, Sim4 and Fta1 are members of a class of kinetochore proteins found specifically in fungi and metazoa with regional *CEN*s and not in fungi with point *CEN*s.

In contrast to CenH3^CENP-A^, CENP-C and CENP-H, potential orthologs of the human CENP-E, Rod, Zwint and Zwilch proteins were not found in any of the fungi examined. The apparent absence of a fungal Rod or Zwilch is particularly interesting, since their binding partner at human kinetochores, Zw10, has a potential ortholog in *S. cerevisiae*, Dsl1 [55]. Both hsZw10 and scDsl1 play a role in membrane trafficking during interphase [56], but scDsl1 is not known to localize to kinetochores. Thus, whereas human hsZw10 functions in vesicle-MT and chromosome-MT interaction, scDsl1 appears to have only the former function, presumably because Rod and Zwilch are not present. The absence of Zw10 from fungal kinetochores is also sufficient to explain the absence of Dynein: the Rod/Zw10/Zwilch (RZZ) complex is needed for the association of Dynein with human and *Drosophila *kinetochores [57]. Considering these data together, we conclude that animal cell kinetochores contain proteins, currently comprising perhaps 25% of the total (and likely to increase), that are absent in fungi with either regional or point *CEN*s.

### Evolutionary relationships among kinetochores

**Figure 8:**
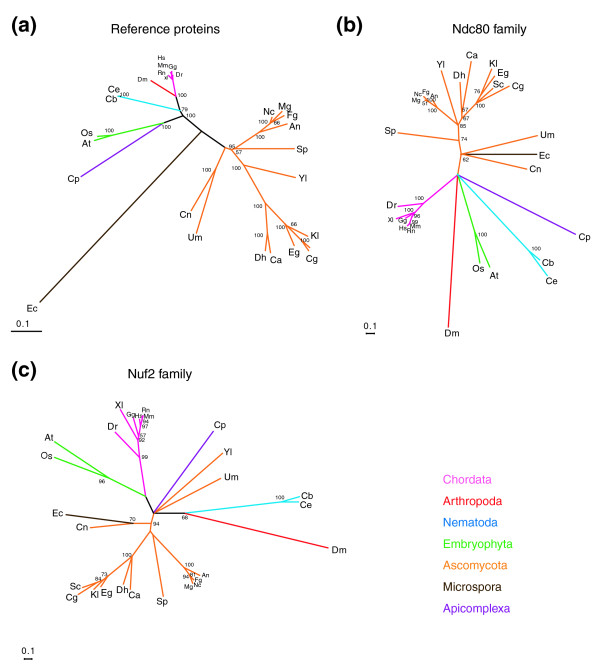
Phylogenetic analysis of kinetochore protein conserved domains. Radial phylogenetic trees were assembled for **(a) **reference proteins (α-tubulin, the signal recognition protein SRP54 and the DNA replication factor PCNA), **(b) **the Ndc80 family and **(c) **the Nuf2 family. For bootstrap analysis, sample size equals 100. Nodes with support less than 50% were collapsed. The accession number for each protein is described in Additional data file 1.

**Figure 9:**
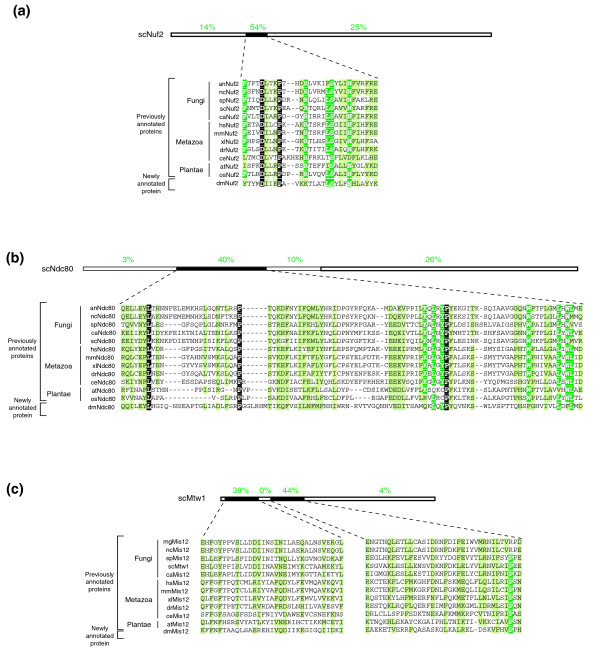
Identification and annotation of **(a) **Nuf2, **(b) **Ndc80 and **(c) **Mis12 orthologs in *D. melanogaster*. Schematic drawing above the alignment indicate the length of the *S. cerevisiae *proteins and the percentages denote the degree of similarity of successive sequence blocks (black boxes). White letters on black denote identical residues, white letters on green, identical residues in ≥ 80% of the organisms and black letters on green, similar residues in ≥ 80% of the organisms. Accession numbers are described in Additional data file 1.

Thus far, we have distinguished only between point and regional *CEN*s but a more nuanced view can be obtained from phylogenetic analysis of kinetochore structural proteins. As a reference for these comparisons, a tree was constructed by combining data on three well-conserved eukaryotic proteins: α-tubulin, PCNA and SRP54 (Figure 8a; this reference tree closely matches reference trees constructed by others [58,59]). The reference tree exhibited prototypical clustering of fungi in one branch and metazoa in another so that *Drosophila *and *C. elegans *were much closer to humans than *S. pombe *or *S. cerevisiae*. However, the phylogenetic trees for Ndc80^Hec1 ^and Nuf2 were remarkably different: overall sequence divergence was much greater and *Drosophila *and *C. elegans *Ndc80^Hec1 ^(or Nuf2) proteins were not significantly more similar to their human than their fungal counterparts (Figure 8b, c). *Drosophila *Ndc80 and Nuf2 were particularly striking in occupying a branch of the phylogenetic tree distant from all other animals. This great divergence in *Drosophila *kinetochore protein sequence is also illustrated by the fact that, apart from regulatory components, such as the Mad-Bub proteins and a few MAPs, only a limited number of structural kinetochore proteins have been identified in flies (for example, CENP-C [60], CenH3^CID ^[61], the RZZ complex [62], Ndc80^Hec1^, Nuf2, and Mis12^Mtw1 ^;Figure 9).

### Organization of the simplest kinetochore

**Figure 10:**
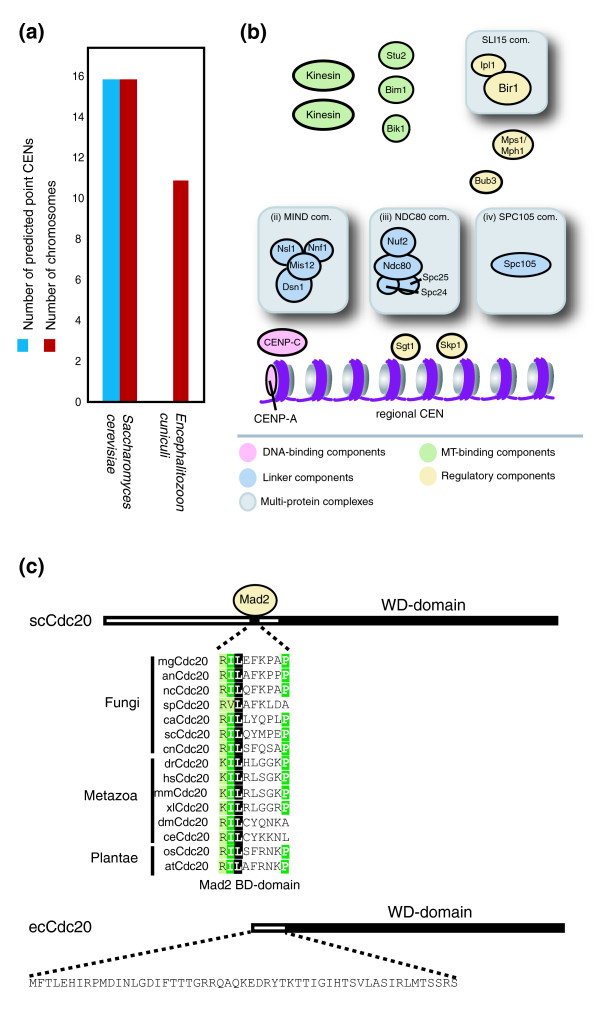
Identification of a minimal kinetochore in *E. cuniculi*. **(a) **HMM described in Figure 1a, b failed to find a CDEI-II-III structure in the genome of *E. cuniculi*. The Green bar indicates point *CEN*s identified and black bars the number of chromosomes. **(b) **Speculative model of *E. cuniculi *kinetochore subunit organization. Proteins colored in pink, blue, green or yellow represent components of the DNA binding, linker, regulatory or microtubule-binding layers, respectively, based on kinetochore organization in *S. cerevisiae*. Potential multi-protein complexes are highlighted with a grey box. **(c) **Sequence alignment of fungal, metazoan and plantae Cdc20 showing the conserved Mad2 binding site. Note: *E. cuniculi *lacks both the conserved Mad2 binding site and an ortholog of the Mad2 protein. Schematic drawings indicate the length of the *S. cerevisiae *and *E. cuniculi *proteins, the position of the WD-domain (black box) and the position where Mad2 binds.

*Encephalitozoon cuniculi *is a microsporidium and intracellular parasite that has been subjected to considerable evolutionary pressure to reduce its genome to the smallest possible size. As a consequence, *E. cuniculi *and related microsporidia have the smallest known eukaryotic proteome (1,997 potential open reading frames) and many cellular structures in *E. cuniculi *lack redundant and non-essential genes [63]. Using our HMM for CDEI-II-III, no sequences similar to point *CENs *were found on any of the 11 *E. cuniculi *chromosomes, nor were CBF3 proteins found by PSI-BLAST (Figure 10a). We therefore speculate that *E. cuniculi *contains a regional *CEN *of some sort. Orthologs of CenH3 and CENP-C^Mif2 ^are present in *E. cuniculi*, as are all four components of the NDC80 linker complex, three components of MIND and SPC105 (Figure 10b, Additional data file 3). No subunits of COMA, the fourth *S. cerevisiae *linker, were found. Among regulatory proteins, *E. cuniculi *Ipl1/Aurora B and Survivin^Bir1 ^orthologs were present as were Mps1 and Bub3, but not other proteins required for the spindle assembly checkpoint in yeast or human cells (Figure 10b). When Cdc20, an essential activator of the anaphase promoting complex (APC/C) was examined for sequence motifs, further evidence was obtained that *E. cuniculi *lacks a spindle checkpoint. APC/C is an E3 ligase required for the ubiquitination of proteins whose destruction is necessary at the metaphase-anaphase transition [64]. In all eukaryotes examined to date, an activated form of the Mad2 checkpoint protein binds to Cdc20 via a short conserved peptide so as to block Cdc20 from activating APC/C, thereby arresting cells at the metaphase-to-anaphase transition [65,66] (Figure 10c). *E. cuniculi *Cdc20 contains the WD-domain implicated in APC/C interaction but lacks any sequence similar to a Mad2 binding domain (Figure 10c), implying that it is not subject to checkpoint control. From these data we conclude that *E. cuniculi *probably contains a very simple kinetochore, based on a regional *CEN *that contains about one-half the proteins found in *S. cerevisiae*. In contrast, other large multi-protein structures in *E. cuniculi *are only slightly less complex than their higher eukaryotic counterparts. For example, *E. cuniculi *ribosomes are composed of 77 subunits as compared to 84 subunits in *S. cerevisiae*. Symptomatic of the simplicity of the *E. cuniculi *kinetochore is the absence of the vast majority of potential MAPs. Nonetheless, it is significant that the *E. cuniculi *kinetochore contains three of the four linker complexes that appear to form the core of budding yeast and human kinetochores.

## Discussion

Extensive genetic and biochemical experimentation has made *S. cerevisiae *kinetochores the best characterized structures involved in chromosome-MT attachment [5]. *S. cerevisiae *kinetochores contain upwards of 70 protein subunits assembled into 14 or more multi-protein complexes. In this study we used similarity-based sequence searching to ascertain which *S. cerevisiae *kinetochore proteins have orthologs in 15 fungi, 11 metazoa and 2 plants (Additional data file 1) with the overall aim of determining which structural features of *S. cerevisiae *kinetochores have been conserved throughout evolution. The analysis is not as straightforward as might be assumed, because kinetochore proteins are among the most rapidly evolving proteins in the genome [67]. In addition, the structure and sequence of *CEN *DNA has diverged widely from organism to organism. Whereas fungi closely related to *S. cerevisiae *contain 125 to 225 bp *CEN*s with a CDEI-CDEII-CDEIII structure, most other organisms contain much longer regional *CEN*s with few if any conserved sequence elements.

Guided by experimental data on established orthologies in yeast, humans and other organisms, we base most of the conclusions in this paper on the characterization of proteins that share blocks of homologous sequence. In several cases, we also draw inferences from a failure to identify homologous proteins. We recognize that this failure represents a negative result with many potential causes. However, in cases in which a kinetochore protein is conserved among organisms A, B and C whereas a second kinetochore protein is well-conserved only in species A and B and undetectable in C (and multiple related species), a tentative conclusion can be drawn that the second protein is actually absent from C. For example, we find that CBF3, an essential *CEN*-binding protein in *S. cerevisiae*, has orthologs in seven budding yeasts containing *CEN *DNA conforming to a CDEI-CDEII-CDEIII organization but not in organisms with regional *CEN*s. In contrast, other kinetochore proteins similar in their degree of sequence conservation to CBF3 subunits among point *CEN*-containing yeast (approximately 45% to 50% similarity) are found throughout fungi. Thus, we provisionally conclude that CBF3 is present in only fungi with CDEI-CDEII-CDEIII centromeres. Despite the potential for occasional error, our use of both positive and negative findings makes it possible to draw broad conclusions about the organization and possible origins of simple and complex kinetochores that would not be possible based on a more conservative approach.

### Origins of point centromeres

Based on the simple structure of their *CEN*s, it is widely assumed that *S. cerevisiae *kinetochores represent an ancestral structure from which complex regional kinetochores evolved. Several findings in the current work suggest, however, that CDEI-II-III *CEN*s arose in combination with a set of 11 proteins as a specialization of a regional *CEN*. First, all annotated organisms containing point *CEN*s (*S. cerevisiae*, *C. glabrata*, *K. lactis*, *and E. gossypii*) have a common origin in one relatively shallow branch of the fungal phylogenetic tree. Were CDEI-II-III sequences an ancestral *CEN*, the current distribution of regional *CEN*s would require loss of point *CEN*s from multiple independent evolutionary branches. Second, we could obtain no evidence for CDEI-II-III *CEN *DNA or CBF3 proteins in the microsporidium *E. cuniculi*, which is thought to have arisen through an ancient divergence in the fungal kingdom [68].

If the speculation that CDEI-II-III point-*CEN*s evolved from regional *CEN*s is correct, we must consider the possible existence of other short *CEN*s that are also based on sequence-specific DNA binding interactions just not CBF3. By way of precedent, the emergence of CDEI-II-III *CEN*s is coincident with large-scale chromosomal changes that gave rise to the *HMR*, *HML *and *MAT *loci, thereby changing the sexual potential of *S. cerevisiae *and related yeasts [29]. *S. pombe *and its close relatives undergo mating type switching analogous to that in *S. cerevisiae*, but the molecular mechanisms of switching are completely different [69]. Functional analysis of fungi with short uncharacterized *CEN*s will be needed to test the speculation that just as different forms of mating-type switching have developed based on distinct biochemistry, point *CEN*s with structures other than CDEI-II-III might exist.

### Evolution of kinetochore proteins

Sequence comparison reveals that conservation among orthologous kinetochore proteins is invariably restricted to relatively short sequence blocks embedded in longer regions of low sequence similarity. The restriction of sequence similarity to small blocks explains the relative difficulty in finding orthologs and the widespread assumption that yeast and human kinetochores are very different. Henikoff and colleagues [67] have studied the evolutionary divergence of CenH3 and CENP-C^Mif2 ^in some detail and propose that kinetochore proteins are under positive selection in plants and animals as a consequence of meiotic drive by *CEN *DNA during female meiosis. Rapid evolution in protein sequence is most apparent in worms and flies, and in this study we have added only dmNdc80, dmNuf2 and dmMis12 to the list of likely structural *Drosophila *kinetochore proteins. Why the rate of kinetochore protein evolution is so much greater in flies and worms as compared to mammals, plants and fungi remains a mystery but it is reminiscent of data on other key regulators of chromosome segregation. Securin and its protease separase are also highly diverged in *D. melanogaster*: *Drosophila *securin, unlike the human and yeast proteins, consists of two separate gene products, called *three rows *and *pimples*, that interact with an unusually short separase [70]. Moreover, unlike the majority of eukaryotes that utilize an RNA-templated reverse transcriptase to replicate telomeres, *D. melanogaster *uses an alternative mechanism based on transposition of the HeT-A and TART retrotransposable elements [71]. It seems very likely that several distinct classes of kinetochore arose early in evolution. Perhaps surprisingly, fungal kinetochores appear to be as good a model for their human counterparts as kinetochores in organisms such as worms and flies.

For the majority of kinetochore proteins we have little knowledge of their biochemical functions or their structure. It is tempting to speculate that conserved sequence blocks represent protein-protein interaction domains or interaction surfaces under tight evolutionary pressure. However, with very few exceptions (for example, the kinase domains of checkpoint and regulatory proteins and motor domains of kinesins), blocks of conserved sequence do not correspond to recognizable functional domains. This stands in contrast to the situation in nuclear pore complexes, in which highly conserved and recognizable domains correspond to key functional units [72]. The most abundant structural elements in kinetochore proteins are coiled coils, which are known to function in protein-protein association [73] and act as springs and levers [74]. Coiled coils in the budding yeast spindle pole body protein scSpc42 also create a crystalline core involved in spindle pole body duplication [75]. Biochemical and electron microscopy experiments have shown that the heptad repeat domains in all four subunits of the *S. cerevisiae *NDC80 complex associate to form an extended stalk that is linked to two globular heads [32,33]. Whether the stalk is simply a spacer or some sort of mechanical element remains unexplored. Only detailed structural and biochemical experiments, backed up by analysis *in vivo*, will reveal the logic of sequence conservation among kinetochore subunits.

### A conserved molecular core of the kinetochore

**Figure 11:**
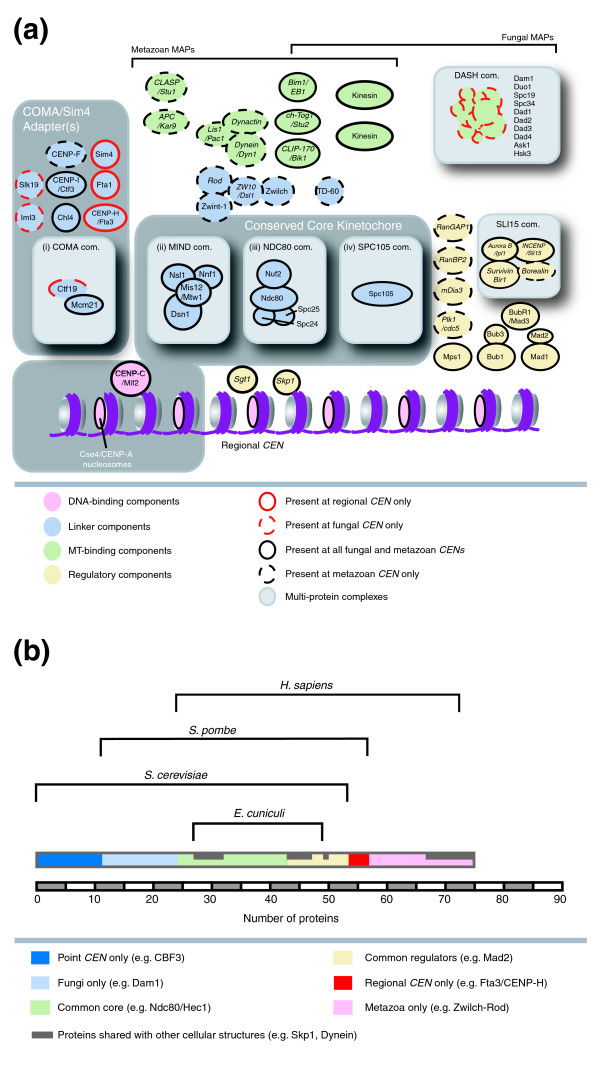
Evolutionary development of kinetochores from yeast to mammals. **(a) **Model of the kinetochore using protein subunit positions derived from the organization of the *S. cerevisiae *kinetochore. Proteins present in all fungal and mammalian *CEN*s are outlined in black while proteins present only in fungi and mammals with regional *CEN*s are outlined in red. Red dotted outlines indicate proteins that are only present in fungi. Black dotted outlines indicate that either this protein only exists in metazoans or that only the metazoan ortholog is present at kinetochore. Proteins colored in pink, blue, green or yellow represent components of the DNA binding, linker, regulatory or microtubule-binding layers, respectively, based on kinetochore organization in *S. cerevisiae*. Potential multi-protein complexes are highlighted with a light gray box and the conserved kinetochore core, or COMA/Sim4 adaptor with a dark gray box. Protein names are given for *H. sapiens *first and then *S. cerevisiae *when different. Italic lettering indicates that the protein has additional functions in the cell. The kinesins present at kinetochores in *H. sapiens *are CENP-E (Kinesin-7) and MCAK (Kinesin-13), and in *S. cerevisiae *Kip3 (Kinesin-8), Cin8 (Kinesin-5), Kip1 (Kinesin-5) and Kar3 (Kinesin-14) (for nomenclature see [38]). **(b) **Quantification of the number of kinetochore proteins, and their respective evolutionary class, in *S. cerevisiae*, *S. pombe*, *E. cuniculi *and *H. sapiens*.

A key conclusion in this paper is that four multi-protein linkers that form the core of the *S. cerevisiae *kinetochore, MIND, the SPC105 complex, the NDC80 complex and COMA are also likely to be present in a wide variety of species (Figure 11). Along with CenH3 and CENP-C, SPC105, MIND and NDC80 complexes are ubiquitous. In budding yeast, linker complexes are thought to form a bridge between proteins in direct contact with DNA and those that bind MTs [5,14,15], and it will be important to show that this is also true in other organisms. Prior to the current work, biochemical experiments had led to the identification of SPC105, MIND and NDC80 complexes in *S. pombe*, *C. elegans *and human cells [18,19] but our systematic sequence analysis extends these observations to a greater variety of organisms, including *E. cuniculi*, a microsporidium with a remarkably small proteome. The presence of the structural kinetochore proteins listed above appears to be more fundamental for chromosome segregation than a Mad2-dependent spindle assembly checkpoint, which does not seem to exist in *E. cuniculi*. Thus, ascertaining the precise molecular functions of the MIND complex, NDC80 complex, Spc105, CenH3 and CENP-C and of the macromolecular assemblies in which they participate is a key task in the study of kinetochore biology.

### Diverged kinetochore components

Budding yeast with CDEI-II-III point *CEN*s contain a set of 11 proteins that are not present in fungi such as *S. pombe *or *C. albicans *(Figure 11). Three of the eleven point-*CEN *specific proteins are involved in sequence-specific binding to CDEIII while six are part of the COMA complex or of a COMA-dependent assembly pathway. Only three of the eleven components of the COMA pathway in *S. cerevisiae *(Mcm21^Mal2^, Chl4^Mis15 ^and Ctf3^Mis6/CENP-I^) are conserved among fungi and mammalian kinetochores (Figure 11). In *S. pombe*, an alternative set of eight proteins, including spSim4 and spFta1-7, are bound to the COMA components spMcm21^Mal2^, spChl4^Mis15 ^and spMis6^Ctf3^. At least three of these proteins (CENP-H^Fta3^, Fta1 and Sim4) are members of a class of proteins found in fungal and metazoan organisms with regional *CEN*s whereas the other four proteins have no obvious orthologs (Figure 11a). Overall, these data point to COMA and COMA-associated proteins as kinetochore components with a particularly high degree of sequence divergence through evolution. It seems reasonable to speculate that COMA helps to accommodate kinetochore subunits that are highly conserved among regional and point *CEN*s, such as the NDC80 complex, to diverged components, such as CBF3. By analogy, it seems likely that specialized proteins have evolved to meet the special structural demands of holocentric *CEN*s; ceKNL-3, a kinetochore protein bound to the *C. elegans *MIND and NDC80 complexes [18] but absent from other kinetochores, may be an early example of a holocentric adaptor.

### The logic of kinetochore assembly

The MT binding components of kinetochores are unlike kinetochore structural components in that almost all are involved in multiple MT-based processes (Figure 11). In humans for example, EB1^Bim1 ^and APC^Kar9 ^are found not only at kinetochores, but also at sites of MT association with the cell cortex; CLIP-170^Bik1 ^and Dynein play important roles in vesicle trafficking and ch-Tog1^Stu2 ^is required for spindle assembly. From yeast to humans, only one or two of the six to ten kinetochore MAPs and motors are specific to kinetochores. CENP-A functions in most organisms to determine *CEN *location without recognizing *CEN*-specific sequences; similarly, the NDC80-MIND-SPC105-COMA complexes must determine the specialized biochemistry of MT-kinetochore linkages without resort to many kinetochore-specific MAPs.

## Conclusion

We conclude that critical structural features of kinetochores are conserved from yeast to man, despite highly divergent *CEN *sequences. It appears that both short *S. cerevisiae *point centromeres and complex metazoan regional centromeres arose from a common ancestor that probably had regional centromeres. Both simple and complex kinetochores contain conserved SPC105, MIND and NDC80 complexes along with more variable COMA complexes. This core assembly is supplemented by adaptor proteins specific to organisms with point, regional or holocentric *CEN*s. The key to understanding kinetochore biology is now to determine how specialized adaptors and conserved core complexes interact with inner centromere components such as CenH3 and CENP-C to assemble structures capable of binding to and regulating microtubules through the recruitment of MAPs and motors.

## Materials and methods

### Sequence-similarity searches

Database searches were performed on NCBI non-redundant and EST databases using PSI-BLAST and BLAST (protein-protein BLAST (blastp) and genomic BLAST (tblastn)) [76]. Pattern searches were performed using ScanProsite [77]. Multiple sequence alignments were built with ClustalW, MUSCLE and T-Coffee and edited by hand [78-80]. Coiled coil predictions were based on the COILS program using a window size of 28 [81]. Human Nnf1R and Fta1R were identified in PSI-BLAST searches using the full-length *S. pombe *Fta1 or *S. cerevisiae *Nnf1 protein sequences as the queries. To identify human Mcm21R, the *S. cerevisiae *Mcm21 protein sequence was first used as a query in PSI-BLAST searches that yielded fungal Mcm21 related proteins. These proteins were assembled in a multiple sequence alignment from which the motif [HYF]- [KRHDENQ]- [VLI]-x- [HYF]- [ST]- [IVL]- [P]-x-x- [IL]-x- [ILV] was derived, and then used in a pattern search to identify metazoan orthologs. To identify orthologs of *S. cerevisiae *Nsl1 and Chl4, *S. pombe *Sim4 or *H. sapiens *CENP-H conserved blocks were first identified. PSI-BLAST searches were carried out using *S. pombe *Sim4, *S. cerevisiae *Nsl1, *S. cerevisiae *Chl4 or *H. sapiens *CENP-H as query sequences. This approach identified a set of fungal Sim4, Nsl1 or Chl4 related proteins and a set of metazoan CENP-H related proteins. Each set of proteins was then assembled into multiple sequence alignments and conserved blocks identified (amino acids 1 to 143 for *U. maydis *Nsl1, 1 to 114 for *S. cerevisiae *Chl4, 341 to 373 for *S. pombe *Sim4 and 224 to 269 for *H. sapiens *CENP-H). The sequences present in these conserved blocks were then used in PSI-BLAST searches to identify new fungal (for CENP-H) or metazoan (for Sim4, Chl4 or Nsl1) proteins.

### Phylogenetic analysis

Phylogenetic alignments were generated with MUSCLE using GBlocks to identify conserved blocks [82]. Conserved blocks were selected only if single positions were conserved in at least 50% of the sequences, with higher stringency at flanking positions (80%). A maximum of eight contiguous non-conserved positions were allowed. The minimum block length was five amino acids. Positions with gaps were allowed only if their number did not exceed 50%. Conserved blocks and the number of positions used for each protein family are described in Additional data file 5. To calculate the distances between sequences we took a maximum likelihood approach using TREE-PUZZLE [83] with the 'Pairwise distance calculation only' option, the Jones-Taylor-Thornton substitution matrix [84] and gamma-distributed rates (eight categories) to account for rate heterogeneity (parameters were estimated from the dataset). A neighbor-joining tree was constructed from the distance matrix with the NEIGHBOR program from the PHYLIP package [85]. Reliability of the dataset was assessed by bootstrap. We generated 100 permutation datasets using the SEQBOOT program from the PHYLIP package. From these 100 datasets we calculated distance matrices and constructed neighbor-joining trees using the parameters described above. TREE-PUZZLE was then used with the 'Consensus of user defined trees' option to generate a consensus tree from all neighbor-joining trees (nodes with support less than 50% were collapsed) [86]. Trees were visualized using the SPLITS TREE tool [87]. Amino acid similarity percentages used in multiple sequence alignments are given in Additional data file 4.

### Hidden Markov model based-modeling

The point *CEN *model was constructed from three different sub-models based on the known structure of point *CEN*s. The first sub-model searched for CDEI-like regions in the query sequence using the [T|G]CA[C|G|T][A|C|G]TG motif. The second sub-model then searched for adjacent CDEII-like AT rich regions. The CDEII region was modeled with a HMM using CDEII from *S. cerevisiae *[88]. For the negative model, *S. cerevisiae *genomic DNA was used (the effect of including *CEN*s in the genomic DNA was disregarded). For both datasets, base transition frequencies were determined and the transition matrix for the HMM was calculated. The quality of the HMM was evaluated by screening annotated budding yeast genomes and assessment with a bit score:



Given the identification of CDEI and CDEII sequence elements, a third sub-model searched for an adjacent CDEIII motif using an expression based on the highly conserved CCGGAA motif. Positive hits were evaluated with the bit score calculated from the CDEII HMM, length distribution, AT length, AT runs and synteny.

## Additional data files

The following additional data are available with the online version of this paper. Additional data file 1 contains accession numbers of all proteins that are used in this study. Additional data file 2 shows the multiple sequence alignment of *S. cerevisiae *Spc34, a subunit of the multi-protein DASH complex, with a set of fungal orthologs and a set of related metazoan proteins (NYD-Sp28 family). Additional data file 3 contains multiple sequence alignments of the *E. cuniculi *kinetochore proteins Ndc80, Nuf2R, Mis12/Mtw1, Nnf1, Spc105 and CENP-C amongst five fungi. Additional data files 4 and 5 list amino acid similarities used in all multiple sequence alignments and homology blocks used in phylogenetic analysis, respectively.

## Supplementary Material

Additional File 1 Accession numbers of all proteins that are used in this study. Click here for file

Additional File 2 Identification of a potential ortholog of the DASH complex subunit Spc34 in humans. *S. cerevisiae *Spc34 was aligned with five fungal and four metazoan sequences. Percentages denote the degree of similarity of successive sequence blocks (black boxes). White letters on black denote identical residues, white letters on green, identical residues in ≤ 80% of the organisms and black letters on green, similar residues in ≤ 80% of the organisms. Accession numbers are described in additional data file 1. Click here for file

Additional File 3 Identification of *E. cuniculi *kinetochore proteins. Multiple sequence alignments of the Ndc80, Nuf2, Nnf1, Mis12^Mtw1^, CENP-C^Mif2 ^and Spc105 proteins amongst five fungi and *E. cuniculi*. Percentages denote the degree of similarity of successive sequence blocks (black boxes). White letters on black denote identical residues, white letters on green, identical residues in ≥ 80% of the organisms and black letters on green, similar residues in ≥ 80% of the organisms. Accession numbers are described in additional data file 1. Click here for file

Additional File 4 Amino acid similarities used in all multiple sequence alignments. Click here for file

Additional File 5 Homology blocks used in phylogenetic analysis. Click here for file

## Figures and Tables

**Table 1:** Sequence similarities among selected fungal kinetochore proteins of point *CEN*

Location	Complex	Protein	Ubiquitous	Point CEN specific	Similarity*	Identity*
DNA-binding	Monomer	Mif2	+		65%	23%
	?	Sgt1	+		74%	28%
	CBF3	Cep3		+	65%	14%
		Ctf13		+	53%	9%
		Ndc10		+	48%	10%
Linker layer	COMA	Mcm21	+		45%	7%
		Ctf19	+		47%	7%
		Ame1		+	45%	9%
		Okp1		+	51%	7%
	MIND	Nnf1	+		67%	14%
		Nsl1	+		69%	15%
	NDC80	Ndc80	+		73%	20%
		Spc24	+		63%	6%
	SPC105	Spc105	+		48%	5%
		Ydr532C		+	52%	6%
	?	Chl4	+		52%	11%
	?	Ctf3	+		51%	6%
	?	Nkp1^†^		+	55%	6%
	?	Nkp2^†^		+	63%	6%
	?	Mcm16		+	52%	7%
	?	Mcm22		+	53%	4%
	?	Iml3^‡^	+		24%	6%
	?	Cnn1		+	40%	4%
MT-binding	DASH	Ask1	+		43%	11%
		Dam1	+		54%	6%
Regulatory	?	Bub3	+		65%	18%
	?	Mad2	+		98%	54%

*As determined from the proteins in *S. cerevisiae*, *C. glabrata*, *E. gossyppii *and *K. lactis*. ^†^Instead of *E. gossypii *the sequences were derived from the very closely related *S. kluyveri*. ^‡^Similarity was determined from proteins of the point *CEN *containing *S. cerevisiae*, *S. kudriavzevii*, *K. waltii *and *S. kluyveri*.
