# Supplementary figures and images for: Phylogenetic and structural analysis of centromeric DNA and kinetochore proteins
Source: Genome Biol. 2006 Mar 22;7(3):R23. doi: 10.1186/gb-2006-7-3-r23 (PMC1557759; doi:10.1186/gb-2006-7-3-r23)

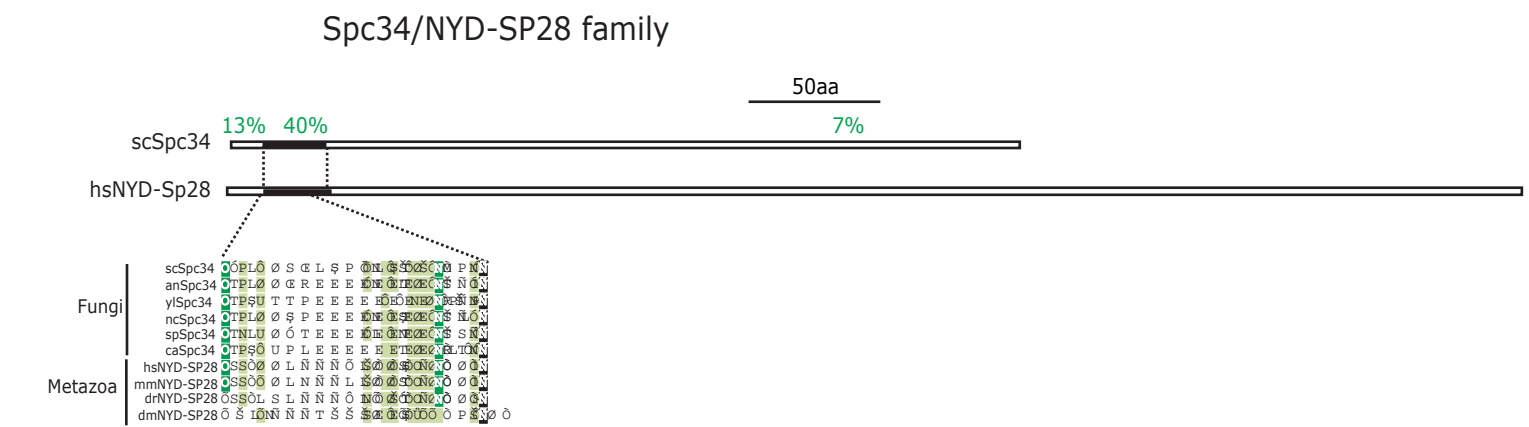

Supplement: Additional File 2 — Identification of a potential ortholog of the DASH complex subunit Spc34 in humans. S. cerevisiae Spc34 was aligned with five fungal and four metazoan sequences. Percentages denote the degree of similarity of successive sequence blocks (black boxes). White letters on black denote identical residues, white letters on green, identical residues in ≤ 80% of the organisms and black letters on green, similar residues in ≤ 80% of the organisms. Accession numbers are described in additional data file 1. [file gb-2006-7-3-r23-S2.pdf]

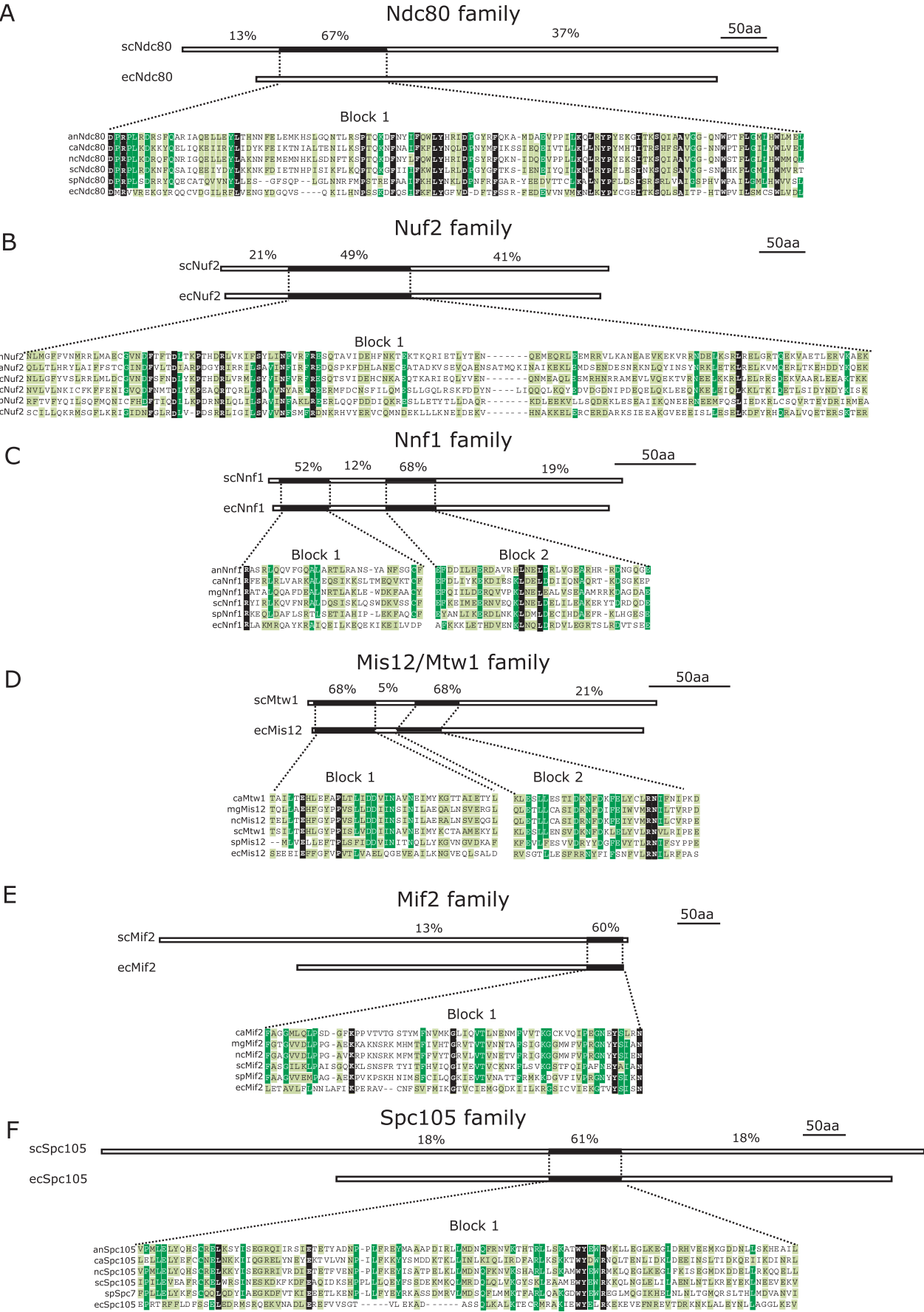

Supplement: Additional File 3 — Identification of E. cuniculi kinetochore proteins. Multiple sequence alignments of the Ndc80, Nuf2, Nnf1, Mis12Mtw1, CENP-CMif2 and Spc105 proteins amongst five fungi and E. cuniculi. Percentages denote the degree of similarity of successive sequence blocks (black boxes). White letters on black denote identical residues, white letters on green, identical residues in ≥ 80% of the organisms and black letters on green, similar residues in ≥ 80% of the organisms. Accession numbers are described in additional data file 1. [file gb-2006-7-3-r23-S3.pdf]
