# Supplementary material for: Phylogenetic and structural analysis of centromeric DNA and kinetochore proteins
Source: Genome Biol. 2006 Mar 22;7(3):R23. doi: 10.1186/gb-2006-7-3-r23 (PMC1557759; doi:10.1186/gb-2006-7-3-r23)
Supplement: Additional File 4 — Amino acid similarities used in all multiple sequence alignments. [file gb-2006-7-3-r23-S4.pdf]

Meraldi et al.
Additional data file 4

Additional data file 4: amino acid similarities

Amino Acid	Similar Amino Acids
A	S, G
C	S
D	E,H, K,N,Q,R
E	D,H, K,N,Q,R
F	H,I,L,V, W,Y
G	A,P,S
H	D,E,F,K,N,Q,R,W,Y
I	F,L,M,V
K	D,E, H,N,Q,R
L	F,I,M,V
M	I,L,V
N	E,D, H,K,Q,R
P	G
Q	D,E, H,K,N,R
R	D,E, H,K,N,Q
S	A,C,G,T
T	S
V	F,I, L,M
W	F,H,Y
Y	F,H,W
