# Supplementary material for: Phylogenetic and structural analysis of centromeric DNA and kinetochore proteins
Source: Genome Biol. 2006 Mar 22;7(3):R23. doi: 10.1186/gb-2006-7-3-r23 (PMC1557759; doi:10.1186/gb-2006-7-3-r23)
Supplement: Additional File 5 — Homology blocks used in phylogenetic analysis. [file gb-2006-7-3-r23-S5.pdf]

Additional data file 5: Conserved Blocks and positions used for phylogenetic analysis		
**Protein**	**Conserved Blocks**	**Number of positions used**
Ndc80	199-204, 206-240, 247-251, 253-267, 269-275, 283-312, 316-328, 477-484, 521-531, 557-581, 586-605, 828-852 and 866-874	201
Nuf2	85-97, 123-141, 222-231, 237-253, 319-330, 334-359, 369-385, 403-412, 489-493, 578-585 and 596-603	145
PCNA	1-6, 20-106, 110-142, 148-197, 300-347 and 363-377	239
SRP54	1-24, 39-65, 86-104, 131-135, 156-365, 380-389, 392-421, 432-457, 515-549, 586-600 and 644-654	412
α-Tubulin	2-9, 21-48 and 61-460	436
